# Psychedelics, epilepsy, and seizures: a review

**DOI:** 10.3389/fphar.2023.1326815

**Published:** 2024-01-12

**Authors:** Ninon Freidel, Liliane Kreuder, Brenden Samuel Rabinovitch, Frank Yizhao Chen, Ryan S. T. Huang, Evan Cole Lewis

**Affiliations:** ^1^ Department of Clinical Research, Numinus Toronto, Toronto, ON, Canada; ^2^ Department of Neuroscience, University of British Columbia Djavad Mowafaghian Centre for Brain Health, Vancouver, BC, Canada; ^3^ Department of Medical Biophysics, University of Toronto Temerty Faculty of Medicine, Toronto, ON, Canada; ^4^ Krembil Research Institute, University Health Network, Toronto, ON, Canada; ^5^ Department of Physiology, University of Toronto Temerty Faculty of Medicine, Toronto, ON, Canada; ^6^ Department of Research, Jamaican Medical Cannabis Corporation, Toronto, ON, Canada; ^7^ Department of Medicine, University of Toronto Temerty Faculty of Medicine, Toronto, ON, Canada; ^8^ Department of Pediatrics, University of Toronto Temerty Faculty of Medicine, Toronto, ON, Canada

**Keywords:** psychedelics, seizures, epilepsy, LSD, psilocybin, magic mushrooms, MDMA, ketamine

## Abstract

Psychedelic compounds have been utilized by humans for centuries for medicinal, religious, and tribal purposes. Clinical trial data starting from the early 2000s and continuing today indicates that psychedelics are a clinically efficacious treatment for a variety of neurological and psychiatric disorders. However, all clinical trials examining these substances have excluded any individual with a past or current history of seizures, leaving a large cohort of epilepsy and non-epilepsy chronic seizure disorder patients without anywhere to turn for psychedelic-assisted therapy. These exclusions were made despite any significant evidence that clinically supervised psychedelic use causes or exacerbates seizures in this population. To date, no clinical trial or preclinical seizure model has demonstrated that psychedelics induce seizures. This review highlights several cases of individuals experiencing seizures or seizure remission following psychedelic use, with the overall trend being that psychedelics are safe for use in a controlled, supervised clinical setting. We also suggest future research directions for this field.

## 1 Introduction

Psychedelic-assisted therapy (PAT) has emerged as a clinically efficacious treatment for an array of psychiatric disorders including treatment-resistant depression (TRD) (Carhart-Harris et al., 2016a; Stroud et al., 2018; Palhano-Fontes et al., 2019; Goodwin et al., 2022), major depressive disorder (MDD) (Davis et al., 2021; Sloshower et al., 2023), end-of-life psychiatric distress (Bossis et al., 2016; Agin-Liebes et al., 2020; Rosa et al., 2022), anxiety (Davis et al., 2019), post-traumatic stress disorder (PTSD) (Mithoefer et al., 2018; Mithoefer et al., 2019) and obsessive-compulsive disorder (OCD) (Rodriguez et al., 2013; Kelmendi et al., 2022). PAT has also effectively treated tobacco addiction and alcohol dependence (Krebs and Johansen, 2012; Bogenschutz et al., 2015; Johnson et al., 2017; Sessa et al., 2019). In these clinical trials, both classical psychedelics, such as psilocybin and lysergic acid diethylamide (LSD), and atypical psychedelics, such as ketamine and 3,4-methylenedioxymethamphetamine (MDMA) were integrated into therapy sessions. These forms of PAT are well-tolerated by patients in clinical settings. Recently, psychedelic treatment has been used to treat functional neurological disorder (FND), an umbrella of neurological symptoms including functional movement disorders (FMDs), functional sensory disorders (FSDs), and functional seizures (Butler et al., 2020; Stewart et al., 2020; Argento et al., 2023).

Although psychedelics have shown strong efficacy in treating a diverse array of symptoms, one specific cohort of patients has been excluded from all PAT clinical trials: individuals with a past or current history of seizures.

Individuals with chronic seizure disorders, such as epilepsy and some mitochondrial encephalopathies, have a reduced quality of life and experience disproportionate rates of anxiety and depression compared to the general population (Carhart-Harris and Nutt, 2017; López-Giménez and González-Maeso, 2018). Additionally, ∼10% of individuals with epilepsy experience functional seizures (also referred to as psychogenic, non-epileptic seizures [PNES]), which are not treatable with traditional anti-epileptic pharmacological therapies or surgical interventions due to their psychological underpinnings. These patients require novel, alternative therapies, with conjunctive psychological treatment, such as in PAT.

There is sparse and conflicting published data regarding the safety of psychedelics in the context of chronic and acute seizures. We will demonstrate that most reports are case studies of individuals taking psychedelics recreationally in unsupervised non-clinical settings. The few controlled studies support classical psychedelics as safe and tolerable under clinical supervision, even in patients with a history of epilepsy who currently experience spontaneous, recurrent seizures (SRS).

The safety profile of classical psychedelics in individuals with epilepsy must be characterized to determine if these compounds are safe for use to treat functional seizures and co-morbid neuropsychiatric conditions. Although this review will focus on epilepsy, the data we present is also relevant to individuals with non-epilepsy chronic seizure disorders, such as mitochondrial encephalopathies. This review aims to summarize the complex mosaic of psychedelics in the context of epilepsy and seizures.

## 2 Methods

### 2.1 Databases and search terms

All data were extracted from public databases including PubMed, Google Scholar, and ResearchGate. The following search terms were used in different combinations: epilepsy, psychedelics, psilocybin, magic mushrooms, mescaline, LSD, lysergide, lysergic acid diethylamide, MDMA, 3,4-methylenedioxymethamphetamine, methylenedioxymethamphetamine, molly, ecstasy, seizures, chronic seizures, acute seizures, serotonergic psychedelics, and hallucinogen.

### 2.2 Screening process

#### 2.2.1 Results screening

Search results were screened by reviewing titles and abstracts. The full text of screened abstracts was reviewed to confirm inclusions.

#### 2.2.2 Inclusion criteria

The inclusion criteria were: 1) any explicit mention of classical and/or atypical psychedelics in the context of acute and/or chronic seizures, and 2) case reports and/or clinical trials involving classical and/or atypical psychedelic use in any seizure disorder or in patients with acute seizures.

## 3 Results

### 3.1 Search results

**FIGURE 1:**
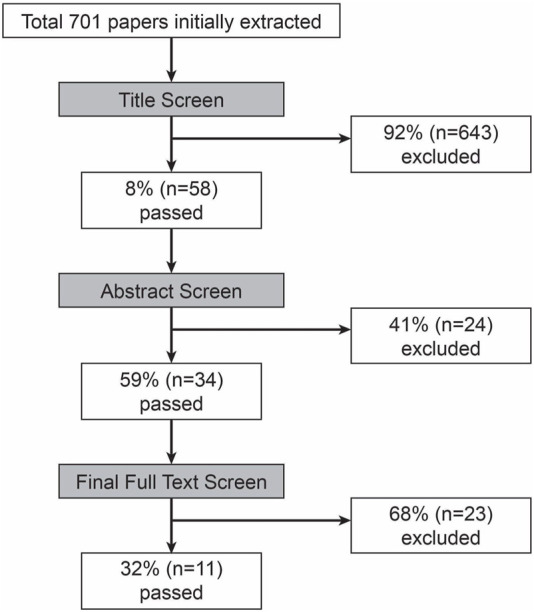
Process of screening papers with initial and final inclusions.

A total of 701 papers were collected. 34 papers passed the title and abstract screens. From there, 11 papers passed the full text screen and were included in the analysis (Figure 1).

### 3.2 Overview of psychedelic actions and mechanisms

#### 3.2.1 Classical psychedelics

The classical psychedelics psilocybin, LSD, and mescaline are agonists of the 5-hydroxytryptamine (5-HT) 2A and 2C receptors (López-Giménez and González-Maeso, 2018). The neural circuitry effects of LSD and psilocybin include changes in resting-state functional connectivity (RSFC) between and within distinct brain regions. Most notably, these compounds increase inter-network RSFC, between the default mode network (DMN), executive network (EN), and salience network (SN) structures, while decreasing intra-network RSFC within each set of structures (Carhart-Harris et al., 2014; Carhart-Harris and Nutt, 2017). The precise neurobiological underpinnings of these functional network changes are still not completely understood.

#### 3.2.2 MDMA

MDMA is an atypical psychedelic, referred to as an empathogen and entactogen (National Academies of Sciences et al., 2022). Although MDMA is serotonergic, it acts via a different mechanism from the classical psychedelics and also possesses some dopaminergic activity (Schenk and Highgate, 2021). MDMA has several mechanisms of action, including increasing presynaptic serotonin release to the synaptic cleft, inhibiting serotonin reuptake at the presynaptic terminal, and some monoamine oxidase inhibition (Kalant, 2001). The subjective effects of MDMA are reported as less hallucinogenic than the classical psychedelics, with increased feelings of empathy and lovingness both outwardly to others and inwardly to oneself (Dumont et al., 2009; Bedi et al., 2010).

#### 3.2.3 Ketamine

Ketamine, an atypical psychedelic and dissociative anesthetic, is an N-methyl-D-aspartate (NMDA) receptor antagonist. The neurobiological mechanisms underlying ketamine’s effects are still being investigated, as ketamine has several mechanisms of action. Ketamine preferentially binds to the NMDA receptors on GABAergic inhibitory interneurons (Gerhard et al., 2020). Ketamine also binds extra-synaptic NMDA receptors on glutamatergic excitatory neurons at lower subanaesthetic doses (Zorumski et al., 2016). At anaesthetic doses, ketamine binds to NMDA receptors on glutamatergic excitatory neurons, inducing overall reduced excitatory transmission, leading to loss of consciousness. Moreover, ketamine may also induce its rapid and persistent anti-depressive effects through its metabolism into hydroxynorketamine (HNK), which is an antagonist of the excitatory ɑ-amino-3-hydroxy-5-methyl-4-isoxazolepropionic acid (AMPA) receptor (Zanos et al., 2016), which glutamate is an agonist of.

### 3.3 Psychedelic use and seizures

We identified 10 case reports from 1992 to 2023 in which patients experienced seizures after ingesting a classical or atypical psychedelic substance. Reports in each subsection are ordered by date (oldest to newest). One additional paper, which was not a case report (Serafetinides, 1965) is also described, though this paper is an open-label trial of LSD in individuals with epilepsy who undergo neurosurgery.

#### 3.3.1 Reports involving MDMA

Hall et al. (1996) reported a 26-year-old paraplegic male experiencing generalized seizures following the ingestion of an ecstasy (MDMA) tablet (of unknown dosage) alongside concurrent alcohol consumption (Hall et al., 1996). He was treated with 50 mg of intravenous diazepam, dantrolene and anaesthesia (thiopentone, alfentanil and suxamethonium), successfully halting the generalized tonic-clonic seizure. Subsequently, the patient developed hypotension, disseminated intravascular coagulation (DIC), acute renal failure, gross rhabdomyolysis, adult respiratory distress syndrome and hepatic failure. Despite the severity of his condition, he was discharged 17 days later and achieved a complete recovery.

Cooper et al. (1997) reported an accidental ingestion of MDMA (of unknown dosage) resulting in febrile convulsion in a 2-year-old female with a history of speech delay (Cooper and Egleston, 1997). At the time, the patient was undergoing treatment with amoxicillin for an upper respiratory tract infection, but the duration of treatment was not specified. The patient displayed agitation, high fever, rapid heart rate and dilated pupils. She was treated with oxygen, rectal paracetamol, and intravenous diazepam resulting in a full recovery without complications. Initially, the mother did not indicate that the patient ingested any substance, until she was questioned about the patient’s abnormal teeth grinding and oculogyric crisis. Following this, the patient’s urine was analyzed, and the findings showed MDMA and MDA (the metabolite of MDMA) presence. The mother then admitted that the patient had accidently ingested MDMA, which delayed the delivery of treatment. The exact timecourse of the urine analysis and treatment was not described, but it was noted that the patient was admitted to the hospital and underwent the aforementioned treatments prior to the parent’s admission (Cooper and Egleston, 1997).

Magee et al. (1998) reported the case of a 17-year-old female who exhibited generalized seizures 5 and 12 h following the ingestion of an ecstasy (MDMA) tablet (of unknown dosage) with concurrent alcohol consumption (Magee et al., 1998). The patient’s symptoms included drowsiness, incoherent speech, hypotension, and reduced urine sodium levels (115 mmol/L). These symptoms indicated severe dilutional hyponatremia leading to secondary seizure and stupor. The significant salt and water loss resulting from vigorous dancing was effectively treated with intravenous isotonic saline, resulting in a complete recovery.

Huntjens et al. (2022) reported accidental MDMA intoxication in a 14-month-old male toddler with an unremarkable medical history (Huntjens et al., 2022). The patient presented to the emergency department with a generalized tonic-clonic seizure. 2.0 mg (0.2 mg/kg) of intraosseous midazolam was administered, but seizures persisted until the patient received 400 mg (42.5 mg/kg) of intravenous levetiracetam, which successfully terminated the status epilepticus. Blood serum analysis revealed an MDMA concentration of 0.48 mg/L^33^.

Pauwels et al. (2023), reported two independent incidents where a young child accidently ingested ecstasy (MDMA) and subsequently began experiencing seizures (Pauwels et al., 2013). *Patient 1* was a 19-month-old male with an unremarkable medical history who was taken to the hospital due to mowing arm gestures, staring, and eye turning. Urine analysis showed 7.8 mg/L 3,4-methylenedioxyamphetamine (MDA) concentration and 183 mg/L MDMA concentration approximately 1–3 h after initial intoxication. The patient was eventually discharged from the hospital without complications.

*Patient 2* was a 20-month-old female with a history of convulsions after a fall on the head. She was taken to the hospital presenting with hyperthermia, tachycardia, and rigidity. Urine analysis revealed a 6 mg/L MDA and 119 mg/L MDMA concentration, along with trace amounts of cocaine (Pauwels et al., 2013). It should be noted, however, that although this report explicitly described trace amounts of cocaine being present in the main text, the table displaying the toxicology results showed a negative result in the cocaine assay, without any urine concentration listed.

#### 3.3.2 Reports involving LSD

Several case studies describe adults experiencing seizures following the recreational use of psychedelics without clinical supervision.

Picker et al. (1992) reported a potential interaction between LSD and fluoxetine, a selective serotonin reuptake inhibitor (SSRI) after a 16-year-old male undergoing 20 mg/day of fluoxetine treatment for approximately 1 year experienced a focal seizure that progressed to a generalized tonic-clonic seizure after ingesting two “blotters” containing LSD at an unknown dosage (Picker et al., 1992). Interestingly, this may have been a case of serotonin syndrome, which is an acute constellation of symptoms caused by excessive serotonin levels. Serotonin syndrome is often reported when different serotonergic drugs are taken concomitantly, such as an SSRI with a large dose of LSD (Boyer and Shannon, 2005; Foong et al., 2018; Francescangeli et al., 2019).

Legriel et al. (2008) reported the case of a 39-year-old male with a history of depression and chronic alcohol abuse (Legriel et al., 2008). The medication history included 75 mg/kg of clomipramine daily to control depression symptoms, which was discontinued after 6 months. The patient reported experiencing a generalized tonic-clonic seizure 3 years prior following ingestion of lysergic acid amine (LSA), which is structurally similar to LSD. The patient was taken to a hospital and experienced mental confusion and mydriasis. Vital signs included a 120 beats/min heart rate, and 185/130 mmHg blood pressure. At the hospital, the patient experienced another seizure that lasted over 10 min, which did cease following intravenous clonazepam administration. The patient was subsequently intubated and mechanically ventilated and was successfully extubated 3 days after admission. The dose of LSA taken was unknown, and the purity was not determined (Legriel et al., 2008).

Aakeroy et al. (2021), reported a male in his late teens with an unremarkable medical history who arrived at the hospital following ingestion of a “blotter” containing LSD and small amounts of N,N-dimethyl tryptamine (DMT), methamphetamine, amphetamine, and MDMA that resulted in the patient experiencing a tonic seizure and other adverse events including vomiting and cyanosis (Aakerøy et al., 2021). Emergency personnel arrived 25 min after the initial onset of symptoms and found the patient in cardiorespiratory arrest. The patient was intubated and received cardiopulmonary resuscitation (CPR) therapy. A comprehensive drug analysis was conducted on a blotter sample identical to the one the patient ingested, which revealed a dosage of 300 μg LSD. Serum and urine samples were collected 3 h after the initial onset of symptoms and LSD was found to have a serum concentration of 4 ng/mL (12.4 nmol/L) and 1.3 ng/mL (4.0 nmol/L) urine concentration (Aakerøy et al., 2021). For comparison, clinical trial LSD dosing is standardized at or around 75 µg, which induces significant mind-altering effects compared to placebo, with observable changes in functional brain activity, detected by functional magnetic resonance imaging (fMRI) (Carhart-Harris et al., 2016b).

##### 3.3.2.1 Reports involving LSD in a clinically-controlled trial

Serafetinides (1965) examined the effect of LSD in 20 individuals with a temporal lobectomy to treat their epileptic seizures. 1 μg/kg LSD was given to patients (orally) 2–3 days before, and 1 month after their temporal lobectomy surgeries (Serafetinides, 1965). Scalp electroencephalograph (EEG) recordings were taken during the LSD administration to determine changes in brain waves and epileptic activity. During the pre-operation recordings, 12 patients had no change in epileptic activity, while 5 had a decrease and 1 had increased epileptic EEG activity. During the post-operation recordings, 17 patients had no change in epileptic activity, while 2 had decreased and 1 had increased epileptic activity. Overall, this study indicates LSD may be safe for use in individuals with epilepsy (Serafetinides, 1965).

#### 3.3.3 Reports involving psilocybin (“magic mushrooms”)

Lastly, a case study by Blond et al. (2023), reported a significant exacerbation in epileptic seizures following the ingestion of a large dose (3.6 g) of psychedelic mushrooms in a 31-year-old male with a history of refractory frontal epilepsy (Blond and Schindler, 2023). In order to treat the unilateral right temporal epilepsy, the patient had been previously implanted with a responsive neurostimulation system (RNS) that improved morbidity although he continued to experience several focal seizures without awareness per week. According to his RNS data, ingestion of psilocybin at high doses (3.6 g) resulted in 32 long episodes (30 s of prolonged epileptiform activity) while ingestion at low doses (1.5 g) did not change baseline seizure frequency (Blond and Schindler, 2023).

#### 3.3.4 Reports involving ketamine

Recently, Argento et al. (2023) reported the case of a 51-year-old female with refractory functional seizures with a daily frequency (Argento et al., 2023). The patient’s medical history included MDD and PTSD. After years of failing behavioural and pharmacological therapies, she enrolled in a ketamine-assisted therapy program. The patient underwent 3 weeks of ketamine-assisted therapy followed by 20 weeks of intermittent therapy and ketamine sessions. During weeks 1, 3, and 4, the patient received 100, 150, and 200 mg of ketamine sublingually, respectively, with an additional 35, 45, and 60 mg intranasal dose, respectively. See Table 1 for the complete KAT treatment regimen. The type of therapy used was psychotherapy by a clinical psychologist specializing in trauma and somatization disorders. Following the treatment, the patient went into remission for the functional seizures, depressive symptoms, and functional movement symptoms (Argento et al., 2023).

**TABLE 1:** KAT treatment regimen and timeline from Argento et al., (2023). Adapted from Argento et al., (2023) Figure 2.

Weeks	Sublingual dose	Intranasal dose	Treatment type	Therapy administered?
**1**	100 mg	30 mg	KAT session	Yes
**2**	-	-	Break	No
**3**	150 mg	45 mg	KAT session	Yes
**4**	200 mg	60 mg	KAT session	Yes
**5**	100 mg	30 mg	Ketamine maintenance session	Yes
**6**	100 mg	30 mg	Ketamine maintenance session	Yes
**7**	100 mg	30 mg	Ketamine maintenance session	Yes
**8**	150 mg	30 mg	Ketamine maintenance session	Yes
**9**	-	-	Break	No
**10**	150 mg	30 mg	Ketamine maintenance session	Yes
**11**	-	-	Break	Yes
**12**	150 mg	30 mg	Break	Yes
**13**	-	-	Interval update	No
**14**	-	-	Break	Yes
**15**	150 mg	30 mg	Ketamine maintenance session	Yes
**16**	150 mg	-	Ketamine maintenance session	Yes
**17**	-	-	Interval update	No
**18**	-	-	Break	No
**19**	150 mg	-	Ketamine maintenance session	Yes
**20**	-	-	Break	No
**21**	-	-	Break	No
**22**	-	-	Break	No
**23**	150 mg	-	Ketamine maintenance session	Yes

## 4 Discussion

### 4.1 Conclusion

**FIGURE 2:**
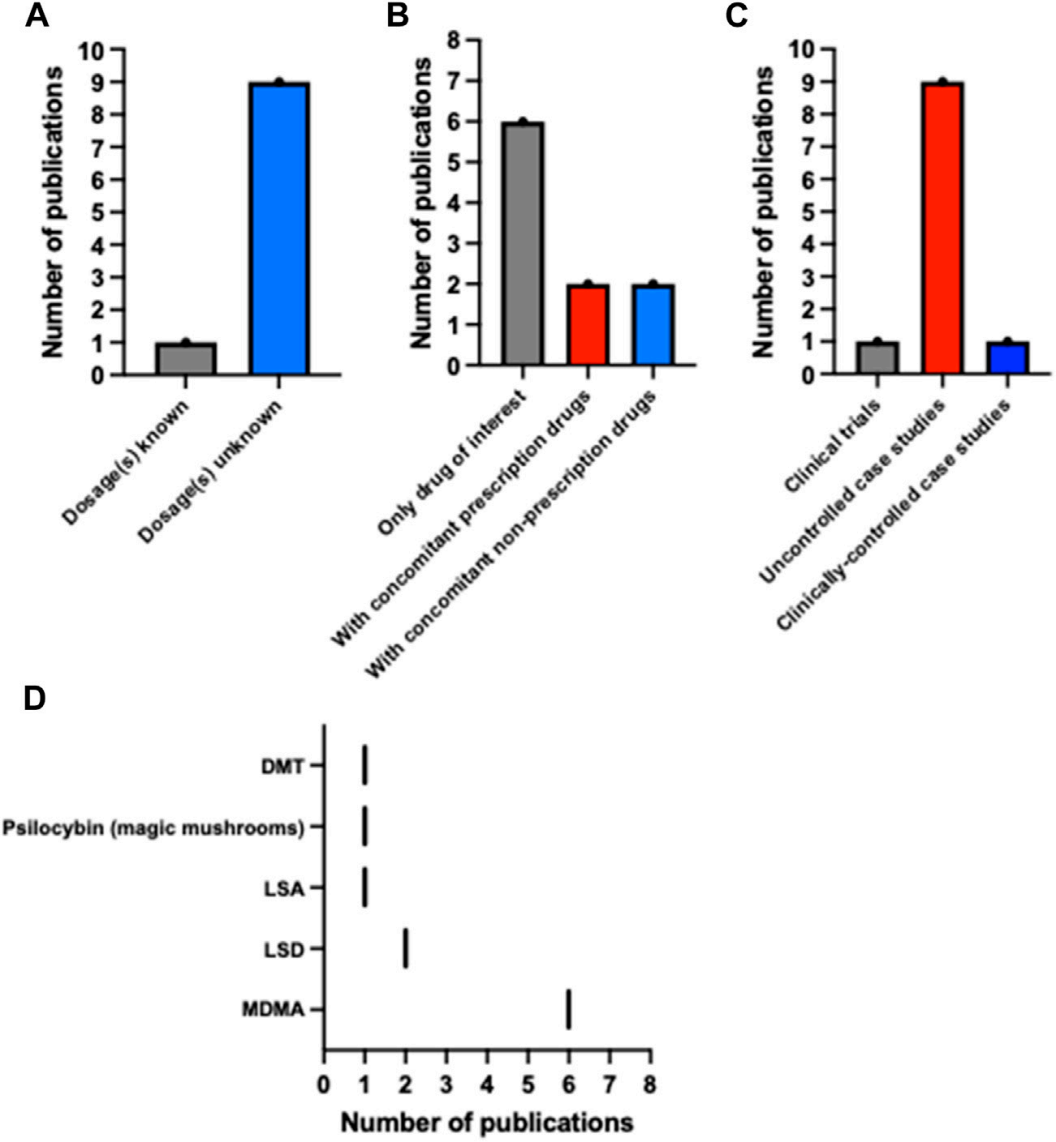
Descriptive statistics of the psychedelic and epilepsy literature. Bar plots of the number of publications describing reports where **(A)** drug dosages were known, **(B)** multiple drugs (prescription and non-prescription) were ingested, and **(C)** there was clinical supervision. **(D)** The number of publications based on the psychedelics ingested in each report. These data highlight variability and sparsity in the literature. Note: Pauwels et al. (2023) counted as n = 2 in this figure due to its reporting of two separate and unrelated cases. **(A,B)** Do not include Serafetinides (1965) due to this study being a clinical trial. This figure was made using GraphPad Prism 10.

In conclusion, there is a deficit of published data on preclinical and clinical uses of psychedelics in the context of epilepsy and non-epilepsy seizure disorders. Most data are case reports of individuals taking unspecified amounts of untested psychedelic drugs in an uncontrolled recreational setting (see Figure 2). Although adverse events were reported, including convulsive seizures, it is unclear whether these events resulted from the psychedelic use, or whether other confounding factors played a role, such as simultaneous alcohol or other drug consumption and concomitant medication, all of which were reported in several cases (see Table 2). Several reports described both accidental and intentional ingestion of other substances, such as cocaine and non-MDMA amphetamines concomitantly with the psychedelic substance (Cooper and Egleston, 1997; Boyer and Shannon, 2005; Huntjens et al., 2022). Additionally, the case reports involving young children ingesting MDMA, though concerning, do not indicate that MDMA induces seizures in individuals with chronic seizure conditions when taken in a controlled clinical setting with appropriate dosing.

**TABLE 2:** Summary of all reports described in the results section. Abbreviations used in this table exclusively: years-old (yo); months-old (mo); generalized tonic-clonic seizure (GTC); not applicable (N/A).

Citation	Drug(s) used	Doses	Age (sex)	Clinical features	Final outcome
**Hall et al. (1996)**	MDMA	Unknown	26yo (male)	- GTC	Complete recovery
				- Hypotension	
				- Disseminated intravascular coagulation (DIC)	
				- Acute renal and hepatic failure	
				- Rhabdomyolysis	
				- Respiratory distress	
**Cooper et al. (1997)**	MDMA, amoxicillin	Unknown	2yo (female)	- Agitation	Complete recovery
				- High fever	
				- Rapid heart rate	
				- Dilated pupils	
**Magee et al. (1998)**	MDMA, alcohol	Unknown	17yo (female)	- GTC	Complete recovery
				- Drowsiness	
				- Incoherent speech	
				- Hypotension	
				- Reduced urine sodium levels (115 mmol/L)	
				- Severe dilutional hyponatremia leading to secondary seizure and stupor	
**Huntjens et al., (2022)**	MDMA	Unknown	14 months (male)	- GTC	Complete recovery
**Pauwels et al. (2023)**	MDMA	Unknown	19 months (male)	- Mowing arm gestures	Complete recovery
				- Staring	
				- Eye turning	
	MDMA	Unknown	20 months (female)	- Convulsions	Complete recovery
				- Hyperthermia	
				- Tachycardia	
				- Rigidity	
**Picker et al. (1992)**	LSD, fluoxetine	- Unknown (LSD); - 20 mg/day (Fluoxetine)	16yo (male)	- Initial focal seizure involving left arm and face that progressed to a GTC	Complete recovery
**Legriel et al. (2008)**	LSA	Unknown	39yo (male)	- GTC	Complete recovery
				- Dilated pupils	
				- Rapid heart rate	
				- Hypertension	
**Aakeroy et al. (2021)**	LSD, DMT, methamphetamine, amphetamine, MDMA	−300 μg (LSD); - unknown (DMT, methamphetamine, amphetamine, MDMA)	Late teens (male)	- GTC	Severe cerebral sequelae 1-year post incident
				- Vomiting	
				- Cyanosis	
				- Cardiorespiratory arrest	
**Serafetinides (1965)**	LSD	1 μg/kg (LSD)	20 patients (12 male, 8 female)	−17 patients had no change in post-operation epileptic activity	N/A
				−2 patients had decreased epileptic activity	
				−1 patient had increased	
**Blond et al. (2023)**	Psilocybin (magic mushrooms)	3.6 g (mushrooms)	31yo (male)	−32 long episodes of prolonged epileptiform activity recorded by implanted responsive neurostimulation system	N/A
**Argento et al. (2023)**	Ketamine	Variable (See Table 1)	51yo (female)	N/A	Remission of symptoms following treatment with ketamine (see subsection *3.3.4*)

It should be noted, however, that in all reports of psychedelic use while under clinical supervision, such as Argento et al. (2023) and Serafetinides (1965), no significant serious adverse events were reported in the individuals who have epilepsy. Although further research is needed, the data we describe in this review indicate that psychedelics may be safe for use in the epilepsy population when taken under clinical supervision in a clinical setting, such as with psychedelic-assisted therapy.

### 4.2 Future research directions

Future research should focus on studying classical psychedelics in preclinical animal and human-derived organoid models of chronic and acute seizures. If it is established that classical psychedelics do not exacerbate seizures in animals, then randomized double-blind placebo-controlled trials should be completed to determine if there is any therapeutic benefit of psychedelics in patients with epilepsy or other chronic seizure disorders to treat their seizures, both epileptic and functional. The field of epilepsy and seizure research is ripe for new data testing classical psychedelic use in acute and chronic seizure disease models. More data is essential to inform clinicians of the potential adverse events or therapeutic benefits of these substances.

## References

[B1] Aakerøy R. Brede G. I. Stolen S. B. Krabseth H. M. Michelsen L. S. Andreassen T. N. (2021). Severe neurological sequelae after a recreational dose of LSD. J. Anal. Toxicol. 45, e1–e3. 10.1093/jat/bkaa145 PMC8363806 33031536

[B2] Agin-Liebes G. I. Malone T. Yalch M. M. Mennenga S. E. Ponté K. L. Guss J. (2020). Long-term follow-up of psilocybin-assisted psychotherapy for psychiatric and existential distress in patients with life-threatening cancer. J. Psychopharmacol. (Oxf.) 34, 155–166. 10.1177/0269881119897615 31916890

[B3] Argento E. Omene E. Jaeger A. H. Kertes A. Mitchell K. A. Necyk C. (2023). Case report: improvement in refractory functional seizures, depression, and quality of life with ketamine-assisted therapy. Front. Neurosci. 17, 1197409. 10.3389/fnins.2023.1197409 37378010 PMC10291615

[B4] Bedi G. Hyman D. de Wit H. (2010). Is ecstasy an ‘empathogen’? Effects of ±3,4-methylenedioxymethamphetamine on prosocial feelings and identification of emotional states in others. Biol. Psychiatry 68, 1134–1140. 10.1016/j.biopsych.2010.08.003 20947066 PMC2997873

[B5] Blond B. N. Schindler E. A. D. (2023). Case report: psychedelic-induced seizures captured by intracranial electrocorticography. Front. Neurol. 14, 1214969. 10.3389/fneur.2023.1214969 37456653 PMC10343433

[B6] Bogenschutz M. P. Forcehimes A. A. Pommy J. A. Wilcox C. E. Barbosa P. C. R. Strassman R. J. (2015). Psilocybin-assisted treatment for alcohol dependence: a proof-of-concept study. J. Psychopharmacol. (Oxf.) 29, 289–299. 10.1177/0269881114565144 25586396

[B7] Bossis A. Ross S. Guss J. Agin-Liebes G. Malone T. Cohen B. (2016). Rapid and sustained symptom reduction following psilocybin treatment for anxiety and depression in patients with life-threatening cancer: a randomized controlled trial. J. Psychopharmacol. (Oxf.) 30, 1165–1180. 10.1177/0269881116675512 PMC5367551 27909164

[B8] Boyer E. W. Shannon M. (2005). The serotonin syndrome. N. Engl. J. Med. 352, 1112–1120. 10.1056/NEJMra041867 15784664

[B9] Butler M. Seynaeve M. Nicholson T. R. Pick S. Kanaan R. A. Lees A. (2020). Psychedelic treatment of functional neurological disorder: a systematic review. Ther. Adv. Psychopharmacol. 10, 2045125320912125. 10.1177/2045125320912125 32435447 PMC7225815

[B10] Carhart-Harris R. Leech R. Hellyer P. J. Shanahan M. Feilding A. Tagliazucchi E. (2014). The entropic brain: a theory of conscious states informed by neuroimaging research with psychedelic drugs. Front. Hum. Neurosci. 8, 20. 10.3389/fnhum.2014.00020 24550805 PMC3909994

[B11] Carhart-Harris R. L. Bolstridge M. Rucker J. Day C. M. J. Erritzoe D. Kaelen M. (2016a). Psilocybin with psychological support for treatment-resistant depression: an open-label feasibility study. Lancet Psychiatry 3, 619–627. 10.1016/S2215-0366(16)30065-7 27210031

[B12] Carhart-Harris R. L. Kaelen M. Bolstridge M. Williams T. M. Williams L. T. Underwood R. (2016b). The paradoxical psychological effects of lysergic acid diethylamide (LSD). Psychol. Med. 46, 1379–1390. 10.1017/S0033291715002901 26847689

[B13] Carhart-Harris R. L. Nutt D. J. (2017). Serotonin and brain function: a tale of two receptors. J. Psychopharmacol. Oxf. Engl. 31, 1091–1120. 10.1177/0269881117725915 PMC5606297 28858536

[B14] Cooper A. J. Egleston C. V. (1997). Accidental ingestion of Ecstasy by a toddler: unusual cause for convulsion in a febrile child. J. Accid. Emerg. Med. 14, 183–184. 10.1136/emj.14.3.183 9193992 PMC1342919

[B15] Davis A. K. Barrett F. S. May D. G. Cosimano M. P. Sepeda N. D. Johnson M. W. (2021). Effects of psilocybin-assisted therapy on major depressive disorder: a randomized clinical trial. JAMA Psychiatry 78, 481–489. 10.1001/jamapsychiatry.2020.3285 33146667 PMC7643046

[B16] Davis A. K. So S. Lancelotta R. Barsuglia J. P. Griffiths R. R. (2019). 5-methoxy-N,N-dimethyltryptamine (5-MeO-DMT) used in a naturalistic group setting is associated with unintended improvements in depression and anxiety. Am. J. Drug Alcohol Abuse 45, 161–169. 10.1080/00952990.2018.1545024 30822141 PMC6430661

[B17] Dumont G. J. H. Sweep F. C. G. J. van der Steen R. Hermsen R. Donders A. R. T. Touw D. J. (2009). Increased oxytocin concentrations and prosocial feelings in humans after ecstasy (3,4-methylenedioxymethamphetamine) administration. Soc. Neurosci. 4, 359–366. 10.1080/17470910802649470 19562632

[B18] Foong A.-L. Grindrod K. A. Patel T. Kellar J. (2018). Demystifying serotonin syndrome (or serotonin toxicity). Can. Fam. Physician 64, 720–727. 30315014 PMC6184959

[B19] Francescangeli J. Karamchandani K. Powell M. Bonavia A. (2019). The serotonin syndrome: from molecular mechanisms to clinical practice. Int. J. Mol. Sci. 20, 2288. 10.3390/ijms20092288 31075831 PMC6539562

[B20] Gerhard D. M. Pothula S. Liu R. J. Wu M. Li X. Y. Girgenti M. J. (2020). GABA interneurons are the cellular trigger for ketamine’s rapid antidepressant actions. J. Clin. Invest. 130, 1336–1349. 10.1172/JCI130808 31743111 PMC7269589

[B21] Goodwin G. M. Aaronson S. T. Alvarez O. Arden P. C. Baker A. Bennett J. C. (2022). Single-dose psilocybin for a treatment-resistant episode of major depression. N. Engl. J. Med. 387, 1637–1648. 10.1056/NEJMoa2206443 36322843

[B22] Hall A. P. Lyburn I. D. Spears F. D. Riley B. (1996). An unusual case of Ecstasy poisoning. Intensive Care Med. 22, 670–671. 10.1007/BF01709744 8844232

[B23] Huntjens D. W. Weersink E. P. S. Hilarius D. L. Ran N. C. Franssen E. J. F. (2022). Severe epileptic seizures after accidental MDMA exposure in a 14-month-old child. Clin. Toxicol. Phila. Pa 60, 657. 10.1080/15563650.2021.1999464 34751607

[B24] Johnson M. W. Garcia-Romeu A. Griffiths R. R. (2017). Long-term follow-up of psilocybin-facilitated smoking cessation. Am. J. Drug Alcohol Abuse 43, 55–60. 10.3109/00952990.2016.1170135 27441452 PMC5641975

[B25] Kalant H. (2001). The pharmacology and toxicology of “ecstasy” (MDMA) and related drugs. CMAJ Can. Med. Assoc. J. 165, 917–928. 11599334 PMC81503

[B26] Kelmendi B. Kichuk S. A. DePalmer G. Maloney G. Ching T. H. W. Belser A. (2022). Single-dose psilocybin for treatment-resistant obsessive-compulsive disorder: a case report. Heliyon 8, e12135. 10.1016/j.heliyon.2022.e12135 36536916 PMC9758406

[B27] Krebs T. S. Johansen P.-Ø. (2012). Lysergic acid diethylamide (LSD) for alcoholism: meta-analysis of randomized controlled trials. J. Psychopharmacol. (Oxf.) 26, 994–1002. 10.1177/0269881112439253 22406913

[B28] Legriel S. Bruneel F. Spreux-Varoquaux O. Birenbaum A. Chadenat M. L. Mignon F. (2008). Lysergic acid amide-induced posterior reversible encephalopathy syndrome with status epilepticus. Neurocrit. Care 9, 247–252. 10.1007/s12028-008-9096-5 18446448

[B29] López-Giménez J. F. González-Maeso J. (2018). Hallucinogens and serotonin 5-ht2a receptor-mediated signaling pathways. Curr. Top. Behav. Neurosci. 36, 45–73. 10.1007/7854_2017_478 28677096 PMC5756147

[B30] Magee C. Staunton H. Tormey W. Walshe J. J. (1998). Hyponatraemia, seizures and stupor associated with ecstasy ingestion in a female. Ir. Med. J. 91, 178. 9973755

[B31] Mithoefer M. C. Feduccia A. A. Jerome L. Mithoefer A. Wagner M. Walsh Z. (2019). MDMA-assisted psychotherapy for treatment of PTSD: study design and rationale for phase 3 trials based on pooled analysis of six phase 2 randomized controlled trials. Psychopharmacol. (Berl.) 236, 2735–2745. 10.1007/s00213-019-05249-5 PMC6695343 31065731

[B32] Mithoefer M. C. Mithoefer A. T. Feduccia A. A. Jerome L. Wagner M. Wymer J. (2018). 3,4-methylenedioxymethamphetamine (MDMA)-assisted psychotherapy for post-traumatic stress disorder in military veterans, firefighters, and police officers: a randomised, double-blind, dose-response, phase 2 clinical trial. Lancet Psychiatry 5, 486–497. 10.1016/S2215-0366(18)30135-4 29728331

[B33] National Academies of Sciences (2022). “History and current status of psychedelics and entactogens for the treatment of psychiatric disorders,” in Exploring psychedelics and entactogens as treatments for psychiatric disorders: proceedings of a workshop (Washington, DC: National Academies Press US). 36049038

[B34] Palhano-Fontes F. Barreto D. Onias H. Andrade K. C. Novaes M. M. Pessoa J. A. (2019). Rapid antidepressant effects of the psychedelic ayahuasca in treatment-resistant depression: a randomized placebo-controlled trial. Psychol. Med. 49, 655–663. 10.1017/S0033291718001356 29903051 PMC6378413

[B35] Pauwels S. Lemmens F. Eerdekens K. Penders J. Poesen K. Desmet K. (2013). Ecstasy intoxication as an unusual cause of epileptic seizures in young children. Eur. J. Pediatr. 172, 1547–1550. 10.1007/s00431-013-2080-x 23828132

[B36] Picker W. Lerman A. Hajal F. (1992). Potential interaction of LSD and fluoxetine. Am. J. Psychiatry 149, 843–844. 10.1176/ajp.149.6.843b 1590506

[B37] Rodriguez C. I. Kegeles L. S. Levinson A. Feng T. Marcus S. M. Vermes D. (2013). Randomized controlled crossover trial of ketamine in obsessive-compulsive disorder: proof-of-concept. Neuropsychopharmacol. Off. Publ. Am. Coll. Neuropsychopharmacol. 38, 2475–2483. 10.1038/npp.2013.150 PMC3799067 23783065

[B38] Rosa W. E. Sager Z. Miller M. Bernstein I. Doerner Rinaldi A. Addicott K. (2022). Top ten tips palliative care clinicians should know about psychedelic-assisted therapy in the context of serious illness. J. Palliat. Med. 25, 1273–1281. 10.1089/jpm.2022.0036 35285721 PMC9467634

[B39] Schenk S. Highgate Q. (2021). Methylenedioxymethamphetamine (MDMA): serotonergic and dopaminergic mechanisms related to its use and misuse. J. Neurochem. 157, 1714–1724. 10.1111/jnc.15348 33711169

[B40] Serafetinides E. A. (1965). The EEG effects of LSD-25 in epileptic patients before and after temporal lobectomy. Psychopharmacologia 7, 453–460. 10.1007/BF00402367 5831880

[B41] Sessa B. Sakal C. O’Brien S. Nutt D. (2019). First study of safety and tolerability of 3,4-methylenedioxymethamphetamine (MDMA)-assisted psychotherapy in patients with alcohol use disorder: preliminary data on the first four participants. BMJ Case Rep. CP 12, e230109. 10.1136/bcr-2019-230109 PMC6663239 31308191

[B42] Sloshower J. Skosnik P. D. Safi-Aghdam H. Pathania S. Syed S. Pittman B. (2023). Psilocybin-assisted therapy for major depressive disorder: an exploratory placebo-controlled, fixed-order trial. J. Psychopharmacol. (Oxf.) 37, 698–706. 10.1177/02698811231154852 36938991

[B43] Stewart B. Dean J. G. Koek A. Chua J. Wabl R. Martin K. (2020). Psychedelic‐assisted therapy for functional neurological disorders: a theoretical framework and review of prior reports. Pharmacol. Res. Perspect. 8, e00688. 10.1002/prp2.688 33280274 PMC7719191

[B44] Stroud J. B. Freeman T. P. Leech R. Hindocha C. Lawn W. Nutt D. J. (2018). Psilocybin with psychological support improves emotional face recognition in treatment-resistant depression. Psychopharmacol. (Berl.) 235, 459–466. 10.1007/s00213-017-4754-y PMC5813058 29085980

[B45] Zanos P. Moaddel R. Morris P. J. Georgiou P. Fischell J. Elmer G. I. (2016). NMDAR inhibition-independent antidepressant actions of ketamine metabolites. Nature 533, 481–486. 10.1038/nature17998 27144355 PMC4922311

[B46] Zorumski C. F. Izumi Y. Mennerick S. (2016). Ketamine: NMDA receptors and beyond. J. Neurosci. 36, 11158–11164. 10.1523/JNEUROSCI.1547-16.2016 27807158 PMC5148235

